# Supplementary material for: Emerging green steel markets surrounding the EU emissions trading system and carbon border adjustment mechanism
Source: Nat Commun. 2025 Oct 13;16:9087. doi: 10.1038/s41467-025-64440-9 (PMC12518805; doi:10.1038/s41467-025-64440-9)
Supplement: Supplementary file 1 — Supplementary information [file 41467_2025_64440_MOESM1_ESM.pdf]

Supplementary information

Emerging green steel markets surrounding the EU Emissions Trading System and Carbon Border Adjustment Mechanism

Constantin Johnson[1], Max Åhman [1*], Lars J. Nilsson [1], Zhenxi Li[1]

[1] Division of Environmental and Energy Systems Studies, Lund university, Box 118, 221 00, Lund, Sweden.

*Corresponding author. E-mail: max.ahman@miljo.lth.se;

Contents

Supplementary Figures **2**

Supplementary Methods **5**

Supplementary Methods 1: Free allocation 5

Supplementary Methods 2: Model input data 7

Supplementary Methods 3: Economics 8

Supplementary Methods 4: Iron ore processing 12

Supplementary Methods 5: Emissions 13

Supplementary Methods 6: Mathematical formulation of the model 14

Supplementary References **21**

Supplementary Figures

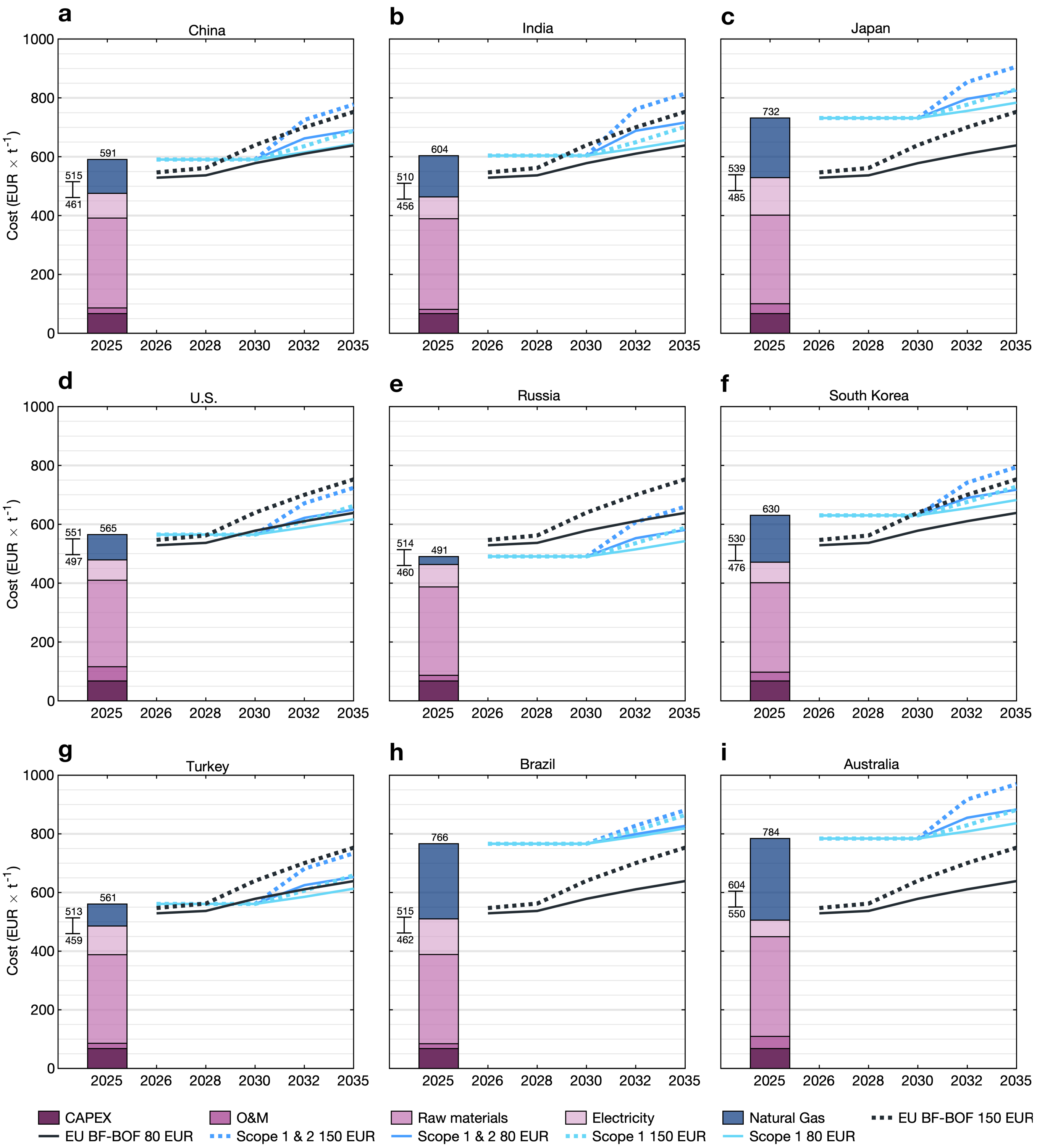

Supplementary Fig. 1: Production costs and total costs of low-emission NG-DRI-EAF. The steel production routes shown are blast furnace–basic oxygen furnace (BF–BOF) and natural gas-direct reduced iron-electric arc furnace (NG-DRI-EAF). **(a-i)** Bars show crude steel production costs without domestic carbon prices and transportation costs. Lines depict total cost which is production cost and CBAM cost excluding domestic carbon prices and transportation costs. The CBAM costs are based on an emission intensity of $1.13\,\mathrm{t\,CO_2\,t\,CS^{-1}}$. Solid lines are total cost with an EU ETS price of €$80\,\mathrm{t\,CO_2^{-1}}$ and dashed lines are total cost with an EU ETS price of €$150\,\mathrm{t\,CO_2^{-1}}$. Domestic BF-BOF production costs are shown to the left with a difference in capital expenditure (CAPEX) between relining and greenfield investments of €$53.8\,\mathrm{tCS^{-1}}$. The total EU BF-BOF costs are shown with the CAPEX for relining.

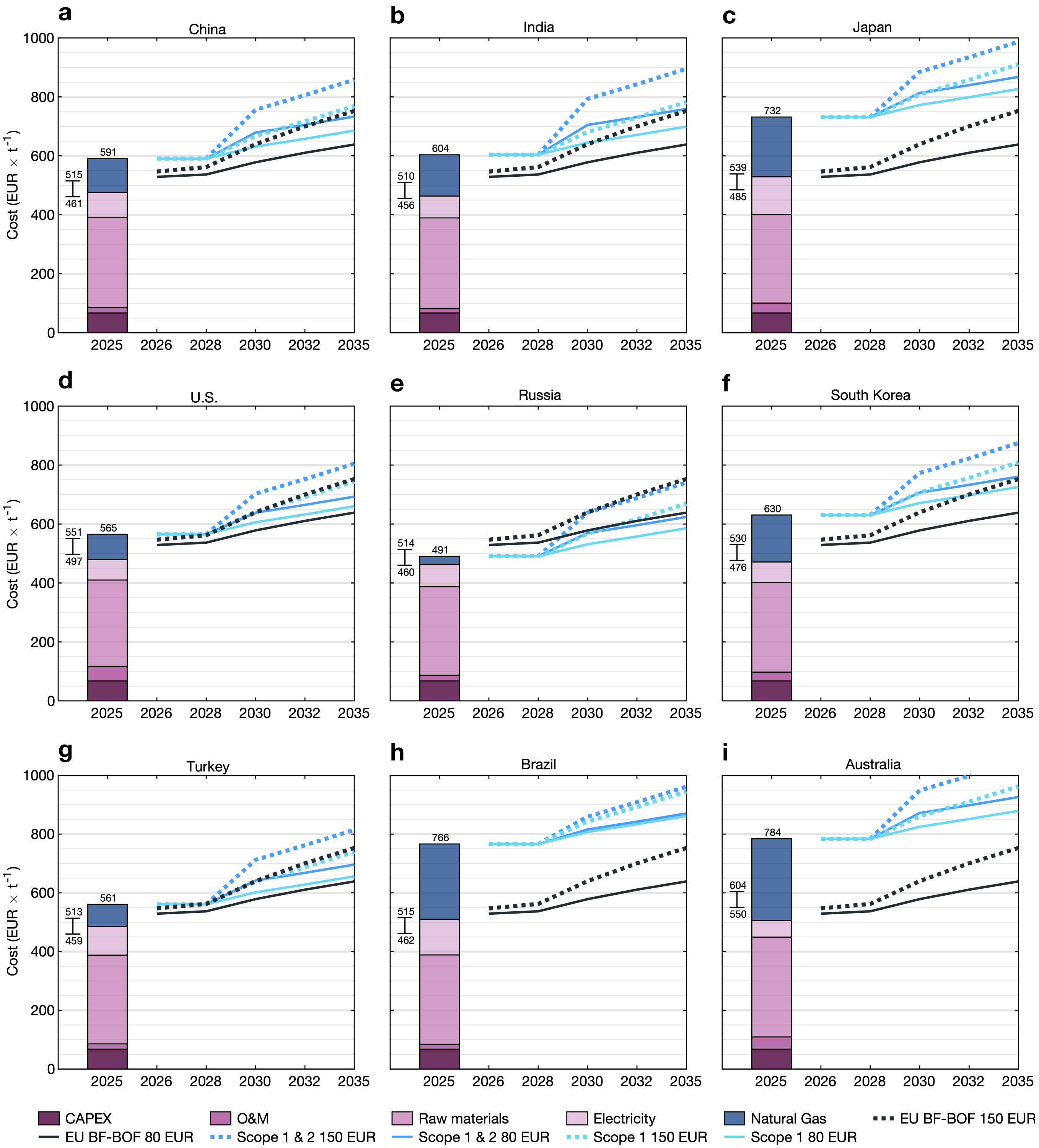

Supplementary Fig. 2: Production costs and total costs of high-emission NG-DRI-EAF. The steel production routes shown are blast furnace–basic oxygen furnace (BF–BOF) and natural gas-direct reduced iron-electric arc furnace (NG-DRI-EAF). **(a-i)** Bars show crude steel production costs without domestic carbon prices and transportation costs. Lines depict total cost which is production cost and CBAM cost excluding domestic carbon prices and transportation costs. The CBAM costs are based on an emission intensity of $1.67\,t\,CO_2\,t\,CS^{-1}$, representing an EU ETS extension to methane based on GWP_{20} and upstream emissions with 3 % leakage. Solid lines are total cost with an EU ETS price of €$80\,t\,CO_2^{-1}$ and dashed lines are total cost with an EU ETS price of €$150\,t\,CO_2^{-1}$. Domestic BF-BOF production costs are shown to the left with a difference in capital expenditure (CAPEX) between relining and greenfield investments of €$53.8\,tCS^{-1}$. The total EU BF-BOF costs are shown with the CAPEX for relining.

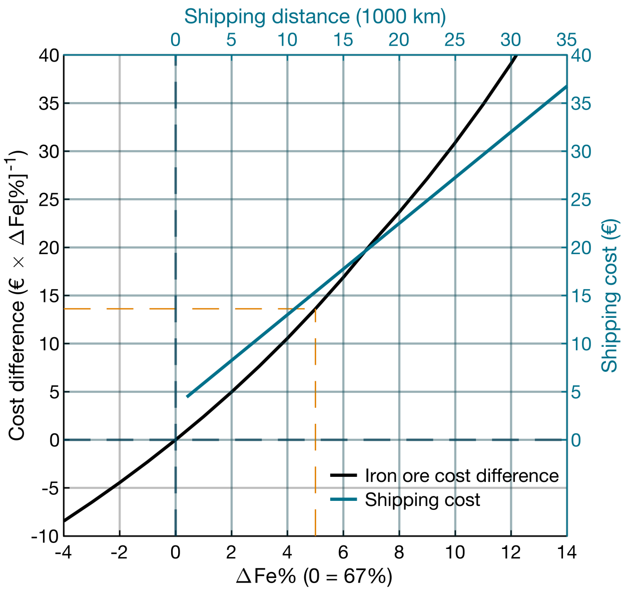

Supplementary Fig. 3: Iron ore processing and shipping costs. Iron ore processing costs as a function of Fe content-difference with direct reduction-grade 67 % as the baseline. Shipping costs as a function of shipping distance. Orange dashed lines indicate the world average Fe content of 62 %.

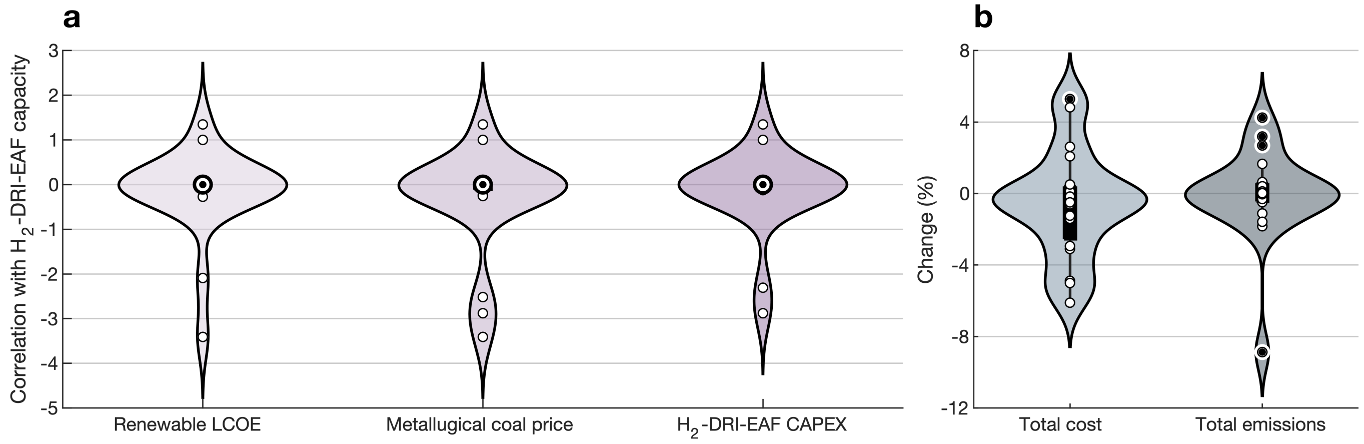

Supplementary Fig. 4: Kernal distribution functions of sensitivity results for ± 20 % parameter changes. a Shows the three largest factors in determining allocated hydrogen-direct reduced iron-electric arc furnace (H_2-DRI-EAF) investments apart from demand reduction. **b** Shows the change to the objective function total cost and cumulative CO_2 emissions.

Supplementary Methods

Supplementary Methods 1: Free allocation

Supplementary Tab. 1: Estimated annual update rates and total update rates for the relevant benchmarks.

Product benchmark	2026-2030	2031-2033	2034-2035
Lime	2.5% (50%)	2.5% (62.5%)	
Coke	2.5% (50%)	2.5% (62.5%)	
Sintered ore	2.5% (50%)	2.5% (57.5%)	-
Hydrogen	2.5% (50%)	2.5% (57.5%)	-
Hot metal	0.3% (6%)	0.3% (6.9%)	-
EAF carbon steel	2.5% (50%)	2.5% (57.5%)	-

The hot metal benchmark has had specific provisions limiting its annual reduction rate to the minimum (Directive 2018/410[1]). This provision has been removed from 2026, but since the 2026-2030 benchmarks are based on year 2021/2022 activity levels and the direct reduced iron produced was minor, the benchmark update for this period will likely remain minimum.

Supplementary Tab. 2: Estimated benchmarks.

Product benchmark	2021-2025	2026-2030	2031-2033	2034-2035
Lime	0.725	0.477	0.358	0.358
Coke	0.217	0.143	0.107	0.107
Sintered ore	0.157	0.086	0.073	0
Hydrogen	6.84	4.425	3.761	0
Hot metal	1.288	1.248	1.236	0
EAF carbon steel	0.215	0.142	0.12	0

The estimated benchmarks from Supplementary Tab. 2 above were adjusted for material consumption for every technological route under study and gives the total amount of free allowances (EUAs) per tonne of produced steel without and with the phase-out (see Supplementary Tabs. 3,4).

Supplementary Tab. 3: Estimated number of free allowances per tonne of crude steel before application of the CBAM factor. The routes blast furnace-basic oxygen furnace (BF-BOF), hydrogen-direct reduced iron-electric arc furnace (H_2-DRI-EAF), and natural gas-direct reduced iron-electric arc furnace (NG-DRI-EAF) have an assumed scrap charge of 12.5 %.

	2021-2025	2026-2030	2031-2033	2034-2035
BF-BOF	1.8326	1.5865	1.4997	0.1802
Scrap-EAF	0.1036	0.1835	0.1518	0.0315
H_2-DRI-EAF	0.0412	1.5851	1.5203	0.0179
NG-DRI-EAF	0.0412	1.3877	1.3524	0.0179

Supplementary Tab. 4: Estimated number of free allowances per tonne of crude steel with application of the CBAM factor. The routes blast furnace-basic oxygen furnace (BF-BOF), hydrogen-direct reduced iron-electric arc furnace (H_2-DRI-EAF), and natural gas-direct reduced iron-electric arc furnace (NG-DRI-EAF) have an assumed scrap charge of 12.5 %.

	**2025**	**2026**	**2027**	**2028**	**2029**	**2030**	**2031**	**2032**	**2033**	**2034**	**2035**
BF-BOF	1.8326	1.5468	1.5072	1.4279	1.2295	0.8170	0.5849	0.3974	0.2100	0.1802	0.1802
Scrap-EAF	0.1036	0.1789	0.1743	0.1651	0.1422	0.0945	0.0592	0.0402	0.0213	0.0315	0.0315
H_2-DRI-EAF	0.0412	1.5455	1.5058	1.4266	1.2285	0.8163	0.5929	0.4029	0.2128	0.0179	0.0179
NG-DRI-EAF	0.0412	1.3530	1.3183	1.2489	1.0755	0.7147	0.5274	0.3584	0.1893	0.0179	0.0179

The preliminary free allocation for steel production for the technologies included in the model were calculated according to Supplementary Equation 1 below.

$$\text{Preliminary free allocation} = \sum_{i=1}^{I}\sum_{n=1}^{N}\left(B_{n,i}\times\text{HAL}_n\times\text{CSCF}_n\times\text{CLEF}_n+B_{n,i}\times\text{HAL}_n\times\text{CSCF}_n\times\text{CLEF}_n\times f^{\text{CBAM}}\right) \quad (1)$$

Where n indicates the type of product benchmark and $i \in [1,3]$ the technology (BF-BOF, scrap-EAF, H_2-DRI-EAF). HAL is the Historical Activity Level, CSCF is the Cross-Sectoral Correction Factor, CLEF is the Carbon Leakage Exposure Factor, and f^{CBAM} is the CBAM factor according to Article 31(1) Regulation 2023/956[2]. The HAL for the periods 2026-2030 and 2031-2035 is determined based on installation data in 2021/2022 and 2026/2027 respectively. Since the CBAM factor is only 5 % in 2027 and the EU ETS cost therefore low, it was assumed that the production volume in 2027 would not be influenced significantly by the phase-out. Thereby, a constant HAL for the years 2026-2035 could be assumed based on the HAL in the period 2021-2025. Furthermore, the amount of free allocation is adjusted if the Actual Activity Level (ALL), i.e. the production volume, deviates from the HAL by 15 %[3], making the assumption of constant HAL based on 2021-2025 an optimistic assumption for competitive BF-BOF production volume. The CSCF is applied if the total demand of all sectors exceeds the possible supply of EAUs (Article 10a(5) Directive 2003/87/EC[4]) and reduces the free allocation for all sectors in a uniform manner except for the 10 % best performing installations[4–6], in this case H_2-DRI-EAF. Due to the oversupply of free allocation in 2021-2024 and the 3 % buffer (Article 10a(5a) Directive 2003/87/EC[4]), along with the updated benchmark values for 2026-2030 resulting in a lower free allocation demand, allows for the assumption of no CSCF activation and can thus be set to one. The CLEF is also set to one due to its definition and the steel sector being at risk of carbon leakage and therefore included in the CBAM. Thereby, the final free allocation can be written according to Supplementary Equation 2 below.

$$\text{Final free allocation} = \sum_{i=1}^{I}\sum_{n=1}^{N}\left(B_{n,i}\times HAL_n^{\text{2021-2025}}+B_{n,i}\times HAL_n^{\text{2021-2025}}\times f^{\text{CBAM}}\right) \quad (2)$$

Supplementary Methods 2: Model input data

Supplementary Tab. 5: Installed capacity used as model input. Numbers from OECD[7], Global Energy Monitor[8], European Steel Association[9], and Ministry of Steel, Government of India[10]. Units in million tonnes (Mt).

Country	BF-BOF	EAF	Total
China	927.086	222.814	1149.9
EU27	100.4974	89.6	190.0874
India	77.1215	43.4515	120.573
Japan	89.73	32.67	122.4
U.S.	37.5	83.4	120.9
Russia	60.8	30.1	90.9
South Korea	53	28.6	81.6
Turkey	14.4	42.8	57.2
Brazil	37.66	12.22	49.88
Australia	4.4	1.41	5.81
Rest of World	246.4475	166.6525	413.1
Total	1 648.6424	753.718	2 425.863

Supplementary Tab. 6: Material consumption for producing one tonne of steel. Units in tonne material per tonne of crude steel or MWh per tonne of crude steel.

Material	BF-BOF	Scrap-EAF	H_2-DRI-EAF	NG-DRI-EAF	Source
Coal	0.78	0	0	0	[11]
Agglomerated iron ore	1.485	0	1.485	1.485	Own calculations based on[11,12]
Scrap	0.1344	1.075	0.1344	0.1344	Own calculations based on[11]
Lime	0.27	0.088	0.05	0.05	[11,12]
Alloys	0.014	0.021	0.011	0.011	Own calculations based on[12,13]
Electricity	0.1153	0.667	1.062	1.062	Own calculations based on[12,14]
Graphite electrodes	0	0.002	0.002	0.002	[12]
Hydrogen	0	0	0.044625	0	Own calculation based on[12]

China's construction of H_2-DRI-EAF capacity was restricted to reflect its national policy of: 1. phasing out obsolete capacity, 2. prohibited new net capacity, and 3. capacity replacement criteria which says that more capacity must be phased out for new capacity to be phased in[15,16]. This is however not a significant limitation to the results of the model since China's production is not below its demand (see Fig. 9) and 35 Mt p.a. of H_2-DRI-EAF capacity exceeds current plans by a vast margin[17].

Supplementary Tab. 7: Maximum added hydrogen-direct reduced iron-electric arc furnace (H_2-DRI-EAF) capacity investments per year. In unit of Mt p.a.

China
35

Supplementary Tab. 8: Maximum assumed import to the EU27 for every type of steel from every country. In unit of Mt p.a.

2022-2025	2026-2030	2031-2035
1.5	5	7

Supplementary Methods 3: Economics

All costs and prices throughout this chapter are presented in EUR 2022.

Supplementary Tab. 9: Capital expenditure (CAPEX) and assumed lifetimes for technology investments. Steel production routes include blast furnace-basic oxygen furnace (BF-BOF), hydrogen-direct reduced iron-electric arc furnace (H_2-DRI-EAF), and natural gas-direct reduced iron-electric arc furnace (NG-DRI-EAF).

Technology	CAPEX [€ t^{-1}]	Lifetime [years]	Source
Relining BF-BOF	64	10.5	[12,18]
Brownfield BF-BOF	227.8	19	[18,19]
Greenfield BF-BOF	592.28	19	[18,19]
EAF	246.56	20	[12,19]
H_2-DRI-EAF	692.24	20	[12,19]
NG-DRI-EAF	660.41	20	[19,20]

Supplementary Tab. 10: Commodities with assumed global market prices.

Commodity	Price [€ t^{-1}]	Source
Metallurgical coal	175	Estimation based on[21].
Lime	119.79	[12]
Alloys	2128.67	[12]
Graphite electrodes	5034.23	[12]

Supplementary Tab. 11: Scrap prices.

Country	Price [€ t^{-1}]	Source
China	373.77	[15]
EU27	381.38	Approximated with Germany[22]
India	454.599	[23]
Japan	308.1	[22]
U.S.	378	[22]
Russia	606.528	[23]
South Korea	424.089	Assumed world average[23]
Turkey	396.576	[23]
Brazil	369.36	[23]
Australia	412.128	[23]
Rest of World	424.089	Assumed world average[23]

Supplementary Tab. 12: Industrial electricity prices.

Country	Price [€ MWh^{-1}]	Source
China	79.2	[24]
EU27	125.4	[25]
India	70	[26]
Japan	120	[27]
U.S.	64.62	[28]
Russia	90	[29]
South Korea	65.9	[30]
Turkey	91.8	[31]
Brazil	114.48	[32]
Australia	234	[29]
Rest of World	181.1	Calculated mean from[31]

Supplementary Tab. 13: Natural gas prices.

Country	Price [€ MWh^{-1}]
China	43[33]
India	52[34]
Japan	75[34]
U.S.	32[34]
Russia	10[34]
South Korea	59[34]
Turkey	28[34]
Brazil	95[34]
Australia	103[35]

Supplementary Tab. 14: Levelised cost of electricity for renewables. All values in unit of € MWh^{-1}.

Country	Utility solar PV	Onshore wind	Offshore wind	Average	Source
China	34.921	25.68	62.766	41.12	[36,37]
EU27	43.75	42	65	50.25	[36,37]
India	35.95	30.5	-	33.225	[36]
Japan	88.33	140	201	143.11	[36]
U.S.	56.49	38.82	-	47.66	[36]
Russia	140	71	-	105.5	[38]
South Korea	82.14	88.37	-	85.255	Own calculations based on[39]
Turkey	65.73	41.9	-	53.815	[36]
Brazil	48.6	21.6	-	35.1	[40]
Australia	38.99	42.86	-	40.93	[37]
Rest of World	49.3	33.9	77	53.4	World average from[36]

Furthermore, the calculated averages from Supplementary Tab. 14 were subjected to an exponential decline according to Supplementary Equation 3.

$$P^{\text{el-renew}}_{j,t_1} = P^{\text{el-renew}}_{j,t_1=2022} \cdot e^{-kt} \;\; \forall\, t \in [0, T-1] \text{ and } \forall\, j \in [1, J] \tag{3}$$

Where $k = 0.015$ for all countries except Japan where $k = 0.04$.

Supplementary Tab. 15: Parameters for calculating shipping costs.

Parameter		Price
Charter rate [€ day^{-1}]	r^c	21 037.5[41]
Average velocity [knt h^{-1}]	$\overline{v}$	14[42]
Fuel consumption at sea [t day^{-1}]	ξ^{sea}	56[42]
Fuel consumption congestion and bunkering [t day^{-1}]	ξ^{con}	1[42]
Fuel price (VLSFO) [€ t^{-1}]	P^{fuel}	612.45[43]
Docking fee [€ visit^{-1}]	c^{har}	179 676[42]
Suez Canal fee [€ t^{-1}]	c^{suez}	2[42]

Supplementary Tab. 16: Data for linear carbon price projections of third countries. If a country is not included its carbon price is assumed equal to zero. Prices in € $t^{-1}_{CO_2}$.

Country	2022	2024	2025	2030	Source
China	0	0	11.241	16.803	[44]
Japan	4.446	-	-	14.4	[45,46]
South Korea	16.191	-	-	25	[47,48]
Turkey	-	22.5	-	45	Own calculations based on[46]

Supplementary Tab. 17: Estimated labour intensities. H_2 production is calculated with an intensity of 2 h kW^{-1}[23] and a 235 W $t^{-1}_{capacity}$ electrolyser (12.5 % scrap charge). Direct reduction (DR) ironmaking labour intensity is based on a labour intensity of 0.22 h t^{-1}_{CS}[23] and adjusted with a metallisation rate of 94 %. Electric arc furnace (EAF) steelmaking is based on a labour intensity of 0.49 h t^{-1}_{CS}[23] and adjusted with a metal yield of 93 %. Labour intensity for blast furnace-basic oxygen furnace (BF-BOF) are own calculations based on Devlin et al.[23] and Mayer et al.[49].

Technology	Labour intensity [h t^{-1}_{CS}]
H_2 production by electrolysis	0.47
DR ironmaking	0.234
EAF steelmaking	0.527
H_2-DRI-EAF	1.181
BF-BOF	1.315

Supplementary Tab. 18: Steelworker hourly rate calculation. Gross National Income (GNI) per capita from The World Bank[50]. Steelworker wages were assumed 30% larger than average and the hourly rates were calculated with 2080 working hours a year. GNI per capita for Rest of World was assumed with 'Middle income'.

**Country**	**GNI per capita [€]**	**Steelworker wage [€]**	**Steelworker hourly rate [€ h^{-1}]**
China	11565	15034.5	7.23
EU27	35708.4	46420.92	22.32
India	2142	2784.6	1.34
Japan	38196	49654.8	23.87
U.S.	68733	89352.9	42.96
Russia	11547	15011.1	7.22
South Korea	32391	42108.3	20.24
Turkey	9531	12390.3	5.96
Brazil	7326	9523.8	4.58
Australia	54387	70703.1	33.99
Rest of World	6379	8292	3.59

Supplementary Tab. 19: Estimated labour costs for the three production routes in the model. Obtained from combining Supplementary Tab. 17 and 18 and in unit of € ${t_{CS}}^{-1}$.

**Country**	**BF-BOF**	**Scrap-EAF**	**H_2-DRI-EAF**
China	9.50	3.81	8.53
EU27	29.34	11.76	26.35
India	1.76	0.71	1.58
Japan	31.39	12.58	28.18
U.S.	56.48	22.63	50.72
Russia	9.49	3.80	8.52
South Korea	26.62	10.67	23.90
Turkey	7.83	3.14	7.03
Brazil	6.02	2.41	5.41
Australia	44.69	17.91	40.12
Rest of World	4.72	1.89	4.24

Supplementary Methods 4: Iron ore processing

For determining the price difference of iron ore based on its Fe-content, a mass flow model to account for process mass losses was constructed as in Supplementary Fig. 5 below.

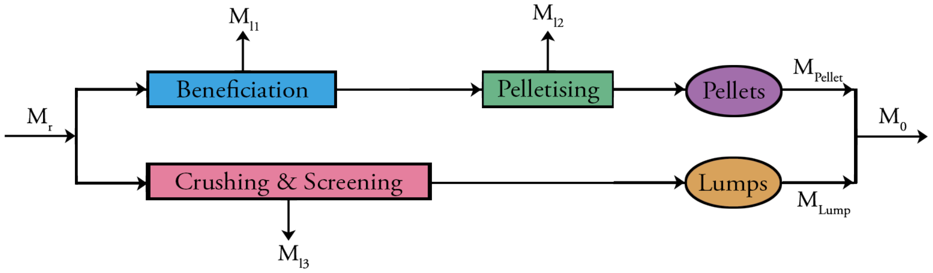

Supplementary Fig. 5: Schematics of mass flow for iron ore processing. The schematics show the mass flow from mined iron ore, run of mine (M_r), to pellets and lumps. Mass losses come from the intermediate processes beneficiation and pelletising for pellets, and crushing and screening for lumps. The Fe-content of the mined ore determines how much beneficiation is necessary to produce pellets with a required Fe-content of 67 %.

The rock mining requirement of run of mine M_r is thus:

$$M_r = \sum_{n=1}^{3} M_{ln} + M_0 \tag{4}$$

However, as a 70%/30% pellet to lump ratio for M_0 was used, Supplementary Equation 4 becomes:

$$M_r = 0.7 \times \left(\frac{M_0}{(1 - M_{l2} \times (0.67 - \mathrm{Fe}_2))} \times \left(\frac{1}{(1 - M_{l1} \times (\mathrm{Fe}_2 - \mathrm{Fe}_1))} + 1 \right) \right) + 0.3 \times \frac{M_0}{(1 - M_{l3} \times (0.67 - \mathrm{Fe}_3))} \tag{5}$$

Where Fe_1 is the iron content from the iron ore that was mined (M_r), Fe_2 is the cut-off Fe-content under which beneficiation is necessary and set to 60% (although arbitrary), and Fe_3 is the cut-off for which Fe-content lumps can be made and set to 66%.

Supplementary Tab. 20: The mass losses used for iron ore processing. Own calculations based on Harvey L. D. D.[51].

Process	Parameter	Mass loss [% $\Delta\mathrm{Fe}^{-1}$]
Beneficiation	M_{l1}	3
Pelletising	M_{l2}	2.78
Crushing & Screening	M_{l3}	19

However, these processes also require energy in the form of electricity and the total energy consumption Π required for DR-grade iron ore products is calculated according to Supplementary Equation 6 below.

$$\begin{aligned} \Pi = {} & 0.7 \times \left(\omega_1 \times \frac{M_0}{(1 - M_{l2} \times (0.67 - \mathrm{Fe}_2)) \times (1 - M_{l1} \times (\mathrm{Fe}_2 - \mathrm{Fe}_1))} \right) + \omega_2 \times M_0 \\ & + \omega_3 \times \left(0.3 \times \frac{M_0}{(1 - M_{l3} \times (0.67 - \mathrm{Fe}_3))} + 0.7 \times \frac{M_0}{(1 - M_{l2} \times (0.67 - \mathrm{Fe}_2))} \right) \end{aligned} \tag{6}$$

The energy consumption parameters can be found in Supplementary Tab. 21 below.

Supplementary Tab. 21: Energy consumption parameters for iron ore processing.

Process	Parameter	Energy consumption [$\text{MWh t}_{product}^{-1}$]
Communition	ω_1	0.02736[23,52]
Pelletising	ω_2	0.20828[23,53]
Crushing & Screening	ω_3	0.00642[23,54]

Combining Supplementary Equation 5 and 6 the total cost for iron ore processing and thus the final cost used as iron ore input to the model is calculated through Supplementary Equation 7.

$$P_j^{io} = P^{\text{ROM}} \times M_0 + P_j^{\text{el-grid}} \times \Pi \tag{7}$$

Where P^{ROM} is the price for run of mine and $P^{\text{el-grid}}$ is the electricity price. P^{ROM} was set to 120 $€\,\text{t}_{\text{ROM}}^{-1}$ for the model which yielded the following iron ore costs for different Fe-contents, which could then be allocated to the different countries according to the Fe-contents from Supplementary Tab. 22.

Supplementary Tab. 22: Fe-content and reserve of domestic iron ore deposits. The reserves are originally from U.S. Geological Survey[55] but adjusted for Fe-content of 67 %. EU27 was approximated with iron ore from the producer LKAB in northern Sweden. Japan and South Korea were disregarded due to insubstantial deposits. Fe-contents are from Devlin et al.[23].

Country	Fe-content [%]	Iron ore reserve [Mt]
China	61	11 311.48
EU27	70	857.14
India	63	5 396.83
U.S.	63	1 587.30
Russia	71	19 718.31
Turkey	63	60.32
Brazil	63	23 809.52
Australia	62	40 322.58
Rest of World	62	200 040.39

Supplementary Methods 5: Emissions

Supplementary Tab. 23: Emission factors for the technologies and countries included in the model. Blast furnace-basic oxygen furnace (BF-BOF) and electric arc furnace (EAF) are from Koolen et al.[56] except Australia and Rest of World which are estimations based on Somers et al.[57] and Hasanbeigi et al.[58]. Hydrogen-direct reduced iron-EAF (H_2-DRI-EAF) emission factors are calculations based on SSAB et al.[59]. All values in unit $\text{t}\,CO_2\,\text{t}_{\text{CS}}^{-1}$.

Country	BF-BOF scope 1	BF-BOF scope 2	EAF scope 1	EAF scope 2	H_2-DRI-EAF scope 1&2
China	1.76	0.08	0.03	0.49	0.064
EU27	1.77	0.04	0.04	0.2	0.064
India	3.72	0.11	0.07	0.38	0.064
Japan	2.05	0.07	0.04	0.36	0.064
U.S.	1.94	0.15	0.04	0.24	0.064
Russia	2.79	0.21	0.07	0.39	0.064
South Korea	2.00	0.05	0.03	0.08	0.064
Turkey	2.17	0.03	0.04	0.25	0.064
Brazil	2.19	0.03	0.05	0.07	0.064
Australia	1.72	0.03	0.06	0.47	0.064
Rest of World	2.238	0.08	0.06	0.38	0.064

Supplementary Methods 6: Mathematical formulation of the model

Supplementary Tab. 24: Sets.

Notation	Description	Range
t	Time	[1,T]
i	Technology	[1,I]
j	Country	[1,J]

Supplementary Tab. 25: Optimisation variables.

Notation	Description
X^{add}	Added steel capacity
Y	Steel production
Z^{steel}	Steel trade
$\Phi^{\text{io-dom}}$	Domestic iron ore use
$\Phi^{\text{io-int}}$	Iron ore trade
$\Psi^{\text{sc-dom}}$	Domestic scrap use
$\Psi^{\text{sc-int}}$	Scrap trade

Supplementary Tab. 26: Cost functions and state variables.

Notation	Description
X^{tot}	Total steel capacity
C_{tot}	Total system cost
C^{Invest}	Investment model cost
$C^{\text{Prod \& Trade}}$	Production system and market model cost
C^{AI}	Total annualised investment cost
C^{OPEX}	Total OPEX cost
C^{ST}	Total steel transportation cost
C^{C}	Total carbon cost
C^{add}	Cost of added capacity
C^{fix}	Total fixed OPEX cost
C^{RES}	Total resource cost
C^{T}	Total resource transportation cost
C^{La}	Total labour cost
$C^{\text{O\&M}}$	Total O&M cost
C^{CBAM}	Total CBAM cost
E^{ship}	Emissions from shipping
E^{sea}	Emissions from sea travel
E^{con}	Emissions from congestion
E^{p}	Emissions from steel production
Ω	Shipping cost
Γ	Total shipping carbon cost

Supplementary Tab. 27: Parameters.

Notation	Description
X^{in}	Installed steel capacity
$\text{CAPEX}^{\text{add}}$	CAPEX for added capacity
r	Discount rate
L	Installation lifetime
f^{CR}	Capital recovery factor
P^{io}	Iron ore price
P^{sc}	Scrap price
P^{c}	Coal price
P^{li}	Lime price
P^{al}	Alloy price
$P^{\text{el-grid}}$	Grid electricity price
$P^{\text{el-renew}}$	Renewable electricity price
P^{ge}	Graphite electrode price
P^{H_2}	Hydrogen price
$P^{\text{EU ETS}}$	EU ETS price
$P^{\text{EU ETS 2}}$	EU ETS 2 price
P^{CM}	Effective carbon price paid by a third country
α	Coal consumption
β	Iron ore consumption
γ	Scrap consumption
δ	Lime consumption
ε	Alloy consumption
ζ	Grid electricity consumption
η	Renewable electricity consumption
θ	Hydrogen consumption
ι	Emission factor
κ	Shipping regression constant
λ	Graphite electrodes
ξ^{sea}	Ship fuel consumption during sea travel
ξ^{con}	Ship fuel consumption during congestion and bunkering
φ	EU ETS 2 phase-in factor
r^{c}	Ship charter rate
d^{sea}	Shipping sea distance
$\overline{v}$	Average ship speed
T^{con}	Congestion and bunkering time
P^{fuel}	Shipping fuel price
c^{har}	Harbour fee
c^{suez}	Suez canal fee
V^{load}	Ship load volume
C^{la}	Labour cost per tonne of steel
m	Mass of input material
B	EU ETS product benchmark
f^{CBAM}	CBAM factor
D	Steel demand
u_{max}	Maximum utilisation rate
r^{growth}	Iron ore deposit annual growth rate
I	Iron ore reserve
S	Scrap availability

The objective of the optimisation model is to minimise the discounted sum of total annual costs of the global steel industry. This is done with two time-sequentially coupled sub-models: an investment model and a production system and market model. Since investment decisions are made regarding future competitiveness and to account for construction time, we adopt a model structure where investment decisions are made τ years in advance of capacity becoming operational. At each time t, the production system and market model is solved for year t, and the investment model is solved for year $t+\tau$, determining added capacity that will become available from year $t+\tau$ onward. Therefore, the production system and market model gains access to the added capacity in year $t+\tau$. Each sub-model minimises the discounted sum of total annual costs. The total system cost is given by Supplementary Equation 8.

$$C_{\text{tot}} = \sum_{t=t_0}^{T} \left(C_{t+\tau}^{\text{Invest}} + C_t^{\text{Prod \& Trade}} \right) \tag{8}$$

The costs in the investment model and the costs in the production system and market model are optimised according to Supplementary Equations 9 and 10:

$$C_{t+\tau}^{\text{Invest}} = \min \left[\left(C_{t+\tau}^{\text{AI}} + C_{t+\tau}^{\text{OPEX}} + C_{t+\tau}^{\text{ST}} + C_{t+\tau}^{\text{C}} \right) \times (1+r)^{-(t+\tau)} \right] \tag{9}$$

$$C_t^{\text{Prod \& Trade}} = \min \left[\left(C_t^{\text{OPEX}} + C_t^{\text{ST}} + C_t^{\text{C}} \right) \times (1+r)^{-t} \right] \tag{10}$$

where $t \in [t_0, T]$. The description of the various components of the objective functions follows below.

The total annual investment cost is calculated according to Supplementary Equation 11:

$$C_{t+\tau}^{\text{AI}} = \sum_{i=1}^{I} \sum_{j=1}^{J} C_{i,j}^{\text{add}} \times X_{t+\tau,i,j}^{\text{add}} \tag{11}$$

where the costs for added capacity are given by Supplementary Equation 12.

$$C_{i,j}^{\text{add}} = f_i^{\text{CR}} \times \text{CAPEX}_{i,j}^{\text{add}} \tag{12}$$

The capital recovery factor in Supplementary Equation 13 utilises technology-specific lifetimes.

$$f_i^{\text{CR}} = \frac{r \times (1+r)^{L_i}}{(1+r)^{L_i} - 1} \tag{13}$$

The total annual cost of operating expenses is given by Supplementary Equation 14:

$$C_t^{\text{OPEX}} = C_t^{\text{fix}} + C_t^{\text{RES}} + C_t^{\text{T}} + C_t^{\text{La}} \tag{14}$$

and the total annual fixed costs of operating expenses is given by Supplementary Equation 15.

$$C_t^{\text{fix}} = \sum_{i=1}^{I} \sum_{j=1}^{J} C_{t,i,j}^{\text{O\&M}} \times X_{t,i,j}^{\text{add}} \tag{15}$$

The total annual cost of resources is given by Supplementary Equation 16.

$$
\begin{aligned}
C_t^{\text{RES}} = & \sum_{j=1}^{J} \Bigg[\sum_{j'=1}^{J} \left[P_j^{\text{io}} \times \left(\Phi_{t,j}^{\text{io-dom}} + \Phi_{t,j,j'}^{\text{io-int}} \right) + P_j^{\text{sc}} \times \left(\Psi_{t,j}^{\text{sc-dom}} + \Psi_{t,j,j'}^{\text{sc-int}} \right) \right] \\
& + \sum_{i=1}^{I} Y_{t,i,j} \times \left(\alpha_i \times P^{\text{c}} + \delta_i \times P^{\text{li}} + \varepsilon_i \times P^{\text{al}} + \zeta_i \times P_j^{\text{el-grid}} + \eta_i \times P_{t,j}^{\text{el-renew}} + \lambda_i \times P_t^{\text{ge}} + \theta_i \times P_{t,j}^{\text{H}_2} \right) \Bigg]
\end{aligned}
\tag{16}
$$

Iron ore and scrap are the only two resources that can be traded in the model and the total annual resource transportation cost is thus given by Supplementary Equation 17:

$$
C_t^{\text{T}} = \sum_{j=1}^{J} \sum_{j'=1}^{J} \left(\Phi_{t,j,j'}^{\text{io-int}} + \Psi_{t,j,j'}^{\text{sc-int}} \right) \times \left(\Omega_{t,j,j'} + \Gamma_{t,j,j'} \right) \tag{17}
$$

where Γ is the carbon cost from EU ETS 2 and Ω is the shipping cost from country j to j' with the sea distance d^{sea} as variable:

$$
\Omega_{t,j,j'} = \frac{r^{\text{c}} \times \left(\kappa_1 + \kappa_2 \times \left(\frac{d_{j,j'}^{\text{sea}}}{\bar{v}} + T^{\text{con}} \right) \right) + P_t^{\text{fuel}} \times \left(\xi^{\text{sea}} \times \left(\kappa_1 - T^{\text{con}} + \kappa_2 \times \left(\frac{d_{j,j'}^{\text{sea}}}{\bar{v}} + T^{\text{con}} \right) \right) + \xi^{\text{con}} \times T^{\text{con}} \right) + c^{\text{har}}}{V^{\text{load}}} + c^{\text{suez}}
$$

κ_1 and κ_2 are the constants obtained from linear regression on real world data and are -5.36 and 1.71 respectively. The carbon costs from EU ETS 2 are applied if the EU is a port of call:

$$
\Gamma_{t,j,j'} = \begin{cases} 0, & t < 4 \\ \frac{1}{2} \times P^{\text{EU ETS 2}} \times \Big(\underbrace{E_{t,j,j'=2}^{\text{ship}}}_{\text{EU import}} + \underbrace{E_{t,j=2,j'}^{\text{ship}}}_{\text{EU export}} \Big), & t \geq 4 \end{cases}
$$

where $t = 4$ represents year 2025, $j, j' = 2$ represents the EU, and E^{ship} are the emissions from shipping:

$$
E_{t,j,j'}^{\text{ship}} = E_{t,j,j'}^{\text{sea}} + E_t^{\text{con}}
$$

which in turn is the sum of emissions from sea travel and congestion and bunkering:

$$
E_{t,j,j'}^{\text{sea}} = \frac{\varphi_t \times \xi^{\text{sea}} \times \left(\kappa_1 - T^{\text{con}} + \kappa_2 \times \left(\frac{d_{j,j'}^{\text{sea}}}{\bar{v}} + T^{\text{con}} \right) \right)}{V^{\text{load}}}
$$

$$
E_t^{\text{con}} = \frac{\varphi_t \times \xi^{\text{con}} \times T^{\text{con}}}{V^{\text{load}}}
$$

The total annual labour costs are given by Supplementary Equation 18.

$$
C_t^{\text{La}} = \sum_{i=1}^{I} \sum_{j=1}^{J} Y_{t,i,j} \times C_{i,j}^{\text{la}} \tag{18}
$$

The total annual transportation cost of steel is given in Supplementary Equation 19:

$$C_t^{\text{ST}} = \begin{cases} \sum_{j=1}^{J}\sum_{j'=1}^{J}\sum_{i=1}^{I} Z_{t,i,j,j'}^{\text{steel}} \times \Omega_{t,j,j'}, & t < 4 \\ \sum_{j=1}^{J}\sum_{j'=1}^{J}\sum_{i=1}^{I} Z_{t,i,j,j'}^{\text{steel}} \times \left(\Omega_{t,j,j'} + \Gamma_{t,j,j'}\right), & t = 4 \\ \sum_{j=1}^{J}\sum_{j'=1}^{J}\sum_{i=1}^{I} Z_{t,i,j,j'}^{\text{steel}} \times \left(\Omega_{t,j,j'} + \Gamma_{t,j,j'} + C_{t,i,j\neq 2}^{\text{CBAM}}\right), & t \geq 5 \end{cases} \tag{19}$$

and the annual carbon cost from the CBAM given by Supplementary Equations 20 and 21:

$$C_{t,i,j\neq 2}^{\text{CBAM}} = \begin{cases} 0, & \text{if } a \leq 0 \quad \text{or } t < 5 \\ a, & \text{if } a > 0 \ \text{ and } t \geq 5 \end{cases} \tag{20}$$

$$a = \left(P_t^{\text{EU ETS}} - P_{t,j\neq 2}^{\text{CM}}\right) \times \left[\iota_{i,j} - \sum_{n=1}^{N}\left(m_{n,i} \times B_{n,i} + m_{n,i} \times B_{n,i} \times f_t^{\text{CBAM}}\right)\right] \tag{21}$$

The total annual carbon costs apart from those borne under the CBAM are given by Supplementary Equation 22:

$$C_t^{\text{C}} = \sum_{i=1}^{I}\sum_{j=1}^{J} P_{t,j}^{\text{CM}} \times E_{t,i,j}^{\text{p}} \tag{22}$$

where the emissions from steel production are given by:

$$E_{t,i,j}^{\text{p}} = \iota_{i,j} \times Y_{t,i,j} \tag{23}$$

The annual carbon cost from the EU ETS is given by Supplementary Equation 24 below.

$$P_{t,j=2}^{\text{CM}} = \begin{cases} 0, & t < 5 \\ P_t^{\text{EU ETS}} \times \left[\sum_{i=1}^{I} Y_{t,i,j=2} \times \left[\iota_{i,j=2} - \sum_{n=1}^{N}\left(m_{n,i} \times B_{n,i} + m_{n,i} \times B_{n,i} \times f_t^{\text{CBAM}}\right)\right]\right], & t \geq 5 \end{cases} \tag{24}$$

Below follows the constraints. The added capacity in year t becomes the lower bound for all years after year t until the end of the investment lifetime:

$$X_{t+1,i,j}^{\text{add}} \geq X_{t,i,j}^{\text{add}} \quad \forall\, t \in [t_0 + \tau, T-1] \ \text{ and } \ \forall\, j \in [1, J] \ \text{ and } \ \forall\, i \in [1, I]$$

Combined with Supplementary Equation 11 this ensures that annualised investment costs are paid throughout the entire investment lifetime. The added capacity is added to the installed capacity X^{in} after a construction time τ and from that year the production system and market model gains access to this added capacity:

$$X_{t,i,j}^{\text{tot}} = X_{t,i,j}^{\text{in}} + X_{t,i,j}^{\text{add}} \quad \forall\, t \in [t_0, T] \ \text{ and } \ \forall\, j \in [1, J] \ \text{ and } \ \forall\, i \in [1, I]$$

The steel demand has to be satisfied at all times while allowing for global trade, hence supply-demand balance dictates that the demand must always be greater or equal than the production plus the import minus the export as shown in Supplementary Equation 25 below.

$$\underbrace{\sum_{i=1}^{I} Y_{t,i,j}}_{\text{Production}} + \underbrace{\sum_{i=1}^{I} \sum_{j'=1}^{J} Z^{\text{steel}}_{t,i,j',j}}_{\text{Import}} - \underbrace{\sum_{i=1}^{I} \sum_{j'=1}^{J} Z^{\text{steel}}_{t,i,j,j'}}_{\text{Export}} \geq \underbrace{D_{t,j}}_{\text{Demand}} \quad \forall\, t \in [1,T] \text{ and } \forall\, j \in [1,J] \tag{25}$$

Additionally, any country can not export more steel than is produced by that country:

$$\underbrace{\sum_{i=1}^{I} \sum_{j'=1}^{J} Z^{\text{steel}}_{t,i,j,j'}}_{\text{Export}} \leq \underbrace{\sum_{i=1}^{I} Y_{t,i,j}}_{\text{Production}} \quad \forall\, t \in [1,T] \text{ and } \forall\, j \in [1,J]$$

The amount of steel produced can not be less than zero and can not exceed the amount of possible steel production, which is determined by the maximum utilisation rate applied to the installed capacity.

$$0 \leq Y_{t,i,j} \leq u_{\max} \times X^{\text{in}}_{t,i,j} \quad \forall\, t \in [1,T],\ \forall\, i \in [1,I] \text{ and } \forall\, j \in [1,J]$$

For the investment model, the following constraint is used:

$$0 \leq Y_{t,i,j} \leq u_{\max} \times X^{\text{tot}}_{t,i,j} \quad \forall\, t \in [1,T],\ \forall\, i \in [1,I] \text{ and } \forall\, j \in [1,J]$$

To reflect reality and since it is not realistic to construct infinite capacity in zero time, a ramp-up rate of 8 % per year was set.

$$\sum_{i=1}^{I} \left(X^{\text{in}}_{t+1,i,j} + X^{\text{add}}_{t+1,i,j} \right) \leq 1.08 \times \sum_{i=1}^{I} \left(X^{\text{in}}_{t,i,j} + X^{\text{add}}_{t,i,j} \right) \quad \forall\, t \in [1,T],\ \forall\, i \in [1,I] \text{ and } \forall\, j \in [1,J]$$

The supply-demand balance for iron ore extraction and trade is given by Supplementary Equations 26-28:

$$\underbrace{\sum_{i=1}^{I} \beta_i \times Y_{t,i,j}}_{\text{Production}} \leq \underbrace{\sum_{j=1}^{J} \Phi^{\text{io-dom}}_{t,j}}_{\text{Domestic}} + \underbrace{\sum_{j'=1}^{J} \Phi^{\text{io-int}}_{t,j',j}}_{\text{Import}} \quad \forall\, t \in [1,T] \text{ and } \forall\, j \in [1,J] \tag{26}$$

$$\underbrace{\Phi^{\text{io-dom}}_{t,j}}_{\text{Domestic}} + \underbrace{\sum_{j'=1}^{J} \Phi^{\text{io-int}}_{t,j,j'}}_{\text{Export}} \leq \underbrace{r^{\text{growth}}_{t} \times I_{t,j}}_{\text{Reserve}} \quad \forall\, t \in [1,T] \text{ and } \forall\, j \in [1,J] \tag{27}$$

$$\underbrace{\sum_{j'=1}^{J} \Phi^{\text{io-int}}_{t,j,j'}}_{\text{Export}} \leq \underbrace{r^{\text{growth}}_{t} \times I_{t,j}}_{\text{Reserve}} \quad \forall\, t \in [1,T] \text{ and } \forall\, j \in [1,J] \tag{28}$$

where r^{growth} is the 3 % CAGR of increased extraction to reflect increasing global steel demand.

The supply-demand balance for scrap utilisation and trade is given by Supplementary Equations 29-31.

$$\underbrace{\sum_{i=1}^{I} \gamma_i \times Y_{t,i,j}}_{\text{Production}} \leq \underbrace{\sum_{j=1}^{J} \Psi_{t,j}^{\text{sc-dom}}}_{\text{Domestic}} + \underbrace{\sum_{j'=1}^{J} \Psi_{t,j',j}^{\text{sc-int}}}_{\text{Import}} \quad \forall\, t \in [1,T] \text{ and } \forall\, j \in [1,J] \tag{29}$$

$$\underbrace{\Psi_{t,j}^{\text{sc-dom}}}_{\text{Domestic}} + \underbrace{\sum_{j'=1}^{J} \Psi_{t,j,j'}^{\text{sc-int}}}_{\text{Export}} \leq \underbrace{S_{t,j}}_{\text{Reserve}} \quad \forall\, t \in [1,T] \text{ and } \forall\, j \in [1,J] \tag{30}$$

$$\underbrace{\sum_{j'=1}^{J} \Psi_{t,j,j'}^{\text{sc-int}}}_{\text{Export}} \leq \underbrace{S_{t,j}}_{\text{Reserve}} \quad \forall\, t \in [1,T] \text{ and } \forall\, j \in [1,J] \tag{31}$$

Supplementary References

1. Directive (EU) 2018/410. *Directive (EU) 2018/410 of the European Parliament and of the Council amending Directive 2003/87/EC to enhance cost-effective emission reductions and low-carbon investments, and Decision (EU) 2015/1814* 2023.
2. Regulation (EU) 2023/956. *Regulation (EU) 2023/956 of the European Parliament and of the Council establishing a carbon border adjustment mechanism* 2023.
3. European Commission. *Guidance Document n° 7 on the harmonised free allocation methodology for the EU ETS post 2020. Guidance on allocation level changes* 2021.
4. Directive 2003/87/EC. *Directive 2003/87/EC of the European Parliament and of the Council establishing a scheme for greenhouse gas emission allowance trading within the Community and amending Council Directive 96/61/EC* Consolidated version 01/03/2024.
5. European Commission. *Guidance Document n° 1 on the harmonised free allocation methodology for the EU ETS – 2024 revision. General Guidance to the allocation methodology* 2024.
6. European Commission. *Guidance Document n° 2 on the harmonised free allocation methodology for the EU ETS – 2024 revision. Guidance on determining the allocation at installation level* 2011.
7. OECD. *OECD Steelmaking Capacity database* 2022. `https://stats.oecd.org/Index.aspx?datasetcode=STI%5C_STEEL%5C_MAKINGCAPACITY`.
8. Global Energy Monitor. *Global Steel Plant Tracker. Operating Steel Capacity (TTPA) by Production Method in Each Country. March 2023 (v2) release.* 2023. `https://globalenergymonitor.org/creative-commons-public-license/`.
9. European Steel Association. *European Steel in Figures 2023* 2023. `https://www.eurofer.eu/assets/publications/brochures-booklets-and-factsheets/european-steel-in-figures-2023/FINAL_EUROFER_Steel-in-Figures_2023.pdf`.
10. Ministry of Steel, Government of India. *Annual report 2022-23* 2023. `https://steel.gov.in/sites/default/files/MoS%5C%20AR%5C%202022-23.pdf`.
11. World Steel Association. *Fact sheet Steel and raw materials* 2023. `https://worldsteel.org/wp-content/uploads/Fact-sheet-raw-materials-2023.pdf`.
12. Vogl, V., Åhman, M. & Nilsson, L. J. Assessment of hydrogen direct reduction for fossil-free steelmaking. *J. Clean. Prod.* **203,** 736–745 (2018).
13. Cappel, J. *EAF Efficiency. AIST virtual MENA steel forum 2021* 2021. `https://www.aist.org/AIST/aist/AIST/Conferences_Exhibitions/MENA/Presentations/AIST_MENA_EAF-Efficiency_Cappel.pdf`.
14. Vidovic, D., Marmier, A., Zore, L. & Moya, J. *Greenhouse gas emission intensities of the steel, fertilisers, aluminium and cement industries in the EU and its main trading partners* JRC134682. Luxembourg, 2023.
15. Li, Z., Andersson, F. N. G., Nilsson, L. J. & Åhman, M. Steel decarbonization in China – a top-down optimization model for exploring the first steps. *J. Clean. Prod.* **384,** 135550 (2023).
16. Ministry of Industry and Information Technology. *Measures for the Implementation of Capacity Replacement in Steel Industry* 2021. `https://www.gov.cn/zhengce/zhengceku/2021-05/07/content_5605092.htm`.
17. LeadIT. *Green Steel Tracker* 2025. `https://www.industrytransition.org/green-steel-tracker/`.
18. Vogl, V., Olsson, O. & Nykvist, B. Phasing out the blast furnace to meet global climate targets. *Joule* **5,** 2646–2662 (2021).
19. BCG *et al. Steel's contribution to a low-carbon Europe 2050* 2013. `https://www.wvstahl.de/wp-content/uploads/Schlussbericht-Studie-Low-carbon-Europe-2050_-Mai-20131.pdf`.
20. IEA. *IEA G20 Hydrogen report: Assumptions* 2020. `https://iea.blob.core.windows.net/assets/29b027e5-fefc-47df-aed0-456b1bb38844/IEA-The-Future-of-Hydrogen-Assumptions-Annex_CORR.pdf`.
21. Trading Economics. 2023. `https://tradingeconomics.com/commodity/coal`.
22. Bureau of International Recycling. *World steel recycling in figures 2018-2022* 2023.

23. Devlin, A., Kossen, J., Goldie-Jones, H. & Yang, A. Global green hydrogen-based steel opportunities surrounding high quality renewable energy and iron ore deposits. *Nat. Commun.* **14,** 2578 (2023).
24. GPP. *Average electricity prices for enterprises in China from September 2019 to March 2023 (in U.S. dollar cents per kilowatt-hour)* In Statista. 2023a. https://www.statista.com/statistics/1373596/business-electricity-price-china/.
25. Ember. *Carbon intensity of the power sector in the European Union in 2022, by country* In Statistia. 2023b. https://www.statista.com/statistics/1291750/carbon-intensity-power-sector-eu-country/.
26. CERC. *Average cost of state electricity supply in India from financial year 2010 to 2022 (in Indian rupees per kilowatt-hour)* In Statista. 2023. https://www.statista.com/statistics/808201/india-cost-of-state-electricity-supply/.
27. METI. *Electricity costs for the industry per kilowatt hour in Japan from fiscal year 2012 to 2021 (in Japanese yen)* In Statistia. 2023. https://www.statista.com/statistics/1220094/japan-electricity-cost-industry/.
28. EIA. *Average retail electricity price for industrial consumers in the United States from 1970 to 2022 (in U.S. dollar cents per kilowatt-hour)* In Statistia. 2023. https://www.statista.com/statistics/190680/us-industrial-consumer-price-estimates-for-retail-electricity-since-1970/.
29. GPP. *Electricity prices for enterprises worldwide in June 2023, by select country (in U.S. dollars per kilowatt-hour)* In Statistia. 2023b. https://www.statista.com/statistics/1369634/business-electricity-price-worldwide-in-selected-countries/.
30. Korea Electric Power Corporation. *System marginal price (SMP) for the electricity market in South Korea from January 2017 to March 2023 (in South Korean won per kilowatt-hour)* In Statistia. 2023. https://www.statista.com/statistics/1388776/south-korea-electricity-market-system-marginal-price/.
31. Department for Energy Security & Net Zero. *Energy Prices International Comparisons. Industrial electricity prices in the IEA.* 2023.
32. EPE. *Average electricity consumption rate in Brazil in 2022, by sector (in Brazilian reals per megawatt-hour)* In Statistia. 2023. https://www.statista.com/statistics/985743/brazil-electricity-consumption-rate-sector/.
33. CEIC. *China Usage Price: 36 City Avg: Natural Gas: Natural Gas for Public Service Sector* 2025. https://www.ceicdata.com/en/china/price-monitoring-center-ndrc-36-city-monthly-avg-transaction-price-production-material/cn-usage-price-36-city-avg-natural-gas-natural-gas-for-public-service-sector.
34. Global Petrol Prices. *Natural Gas Prices* 2024. https://www.globalpetrolprices.com/natural_gas_prices/.
35. Global Petrol Prices. *Australia Natural Gas Prices* 2024. https://www.globalpetrolprices.com/Australia/natural_gas_prices/.
36. IRENA. *Renewable Power Generation Costs in 2021* (Abu Dhabi, 2022).
37. IRENA. *Renewable Power Generation Costs in 2022* (Abu Dhabi, 2023).
38. Filimonova, I., Kozhevin, V., Provornaya, I., Komarova, A. & Nemov, V. Green energy through the LCOE indicator. *Energy Reports* **8,** 887–893 (2022).
39. Choi, D., Eom, J. & Cho, C. Integrating market and technology uncertainties into the projected cost of power technologies: a case of Korea. *Energy Strat. Rev.* **45,** 101046 (2023).
40. IRENA. *Renewable Power Generation Costs in 2023* (Abu Dhabi, 2024).
41. Hellenic shipping news. *Crude Tanker Market Outlook: August 2022* 2022. https://www.%20%5Cnewline%20hellenicshippingnews.com/crude-tanker-market-outlook-august-2022/.
42. Vögele, S., Grajewski, M., Rübbelke, D. & Zobel, L. *Initial Data for Assessment of Cost of Steel Production* doi: 10.17632/6bnj5h9w59.3. 2023.
43. Ship & Bunker. https://shipandbunker.com/prices/av/global/av-glb-global-average-bunker-price%5C#VLSFO.

44. S&P Global. Yin, I. *Commodities 2023: China's carbon market to slow in 2023 as energy security, economy take priority* 2023. https://www.spglobal.com/commodityinsights/en/market-insights/latest-news/energy-transition/011223-chinas-carbon-market-to-slow-in-2023-as-energy-security-economy-take-priority.
45. ICAP. International Carbon Action Partnership. *Japan - Tokyo Cap-and-Trade Program* 2023a. https://icapcarbonaction.com/en/ets/japan-tokyo-cap-and-trade-program.
46. International Monetary Fund. Asia and Pacific Dept. *Climate Change Policy Options* 2022.
47. ICAP. International Carbon Action Partnership. *Korea Emissions Trading Scheme* 2023b. https://icapcarbonaction.com/en/ets/korea-emissions-trading-scheme.
48. Winchester, N. & Reilly, M. J. The economic, energy, and emissions impacts of climate policy in South Korea. *Climate Change Economics* **10** (2019).
49. Mayer, J., Bachner, G. & Steininger, K. W. Macroeconomic implications of switching to process-emission-free iron and steel production in Europe. *J. Clean. Prod.* **210,** 1517–1533 (2019).
50. The World Bank. *GNI per capita, Atlas method (current US$)* 2023. https://data.worldbank.org/indicator/NY.GNP.PCAP.CD.
51. Harvey, L. D. D. From Iron Ore to Crude Steel: Mass Flows Associated with Lump, Pellet, Sinter and Scrap Iron Inputs. *ISIJ Int.* **60,** 1159–1171 (2020).
52. Palacios, J.-L., Fernandes, I., Abadias, A., Valero, A., Valero, A. & Reuter, M. A. Avoided energy cost of producing minerals: The case of iron ore. *Energy Reports* **5,** 364–374 (2019).
53. Lv, W., Sun, Z. & Su, Z. Life cycle energy consumption and greenhouse gas emissions of iron pelletizing process in China, a case study. *J. Clean. Prod.* **233,** 1314–1321 (2019).
54. Norgate, T. & Haque, N. Energy and greenhouse gas impacts of mining and mineral processing operations. *J. Clean. Prod.* **18,** 266–274 (2010).
55. U.S. Geological Survey. *Mineral Commodity Summaries - Iron Ore* 2022. https://pubs.usgs.gov/periodicals/mcs2022/mcs2022-iron-ore.pdf.
56. Koolen, D. & Vidovic, D. *Greenhouse gas intensities of the EU steel industry and its trading partners* JRC129297 (Publications Office of the European Union, Luxembourg, 2022).
57. Somers, J. *Technologies to decarbonise the EU steel industry* JRC127468 (Publications Office of the European Union, Luxembourg, 2022).
58. Hasanbeigi, A. & Springer, C. *How Clean is the U.S. Steel Industry? An International Benchmarking of Energy and CO2 Intensities.* (San Francisco CA, 2019).
59. SSAB, LKAB & Vattenfall. *Fossilfri stålproduktion redo för industrialisering* 2024. https://www.hybritdevelopment.se/wp-content/uploads/2023/08/hybrit-rapport-svenska.pdf.
